# Hyperuricemia and uncontrolled hypertension in treated hypertensive patients: K-MetS Study

**DOI:** 10.1097/MD.0000000000004177

**Published:** 2016-07-18

**Authors:** Jaelim Cho, Changsoo Kim, Dae Ryong Kang, Jeong Bae Park

**Affiliations:** a Department of Occupational and Environmental Medicine, Gachon University Gil Medical Center, Incheon; b Department of Preventive Medicine, Yonsei University College of Medicine, Seoul; c Department of Humanities and Social Medicine, Ajou University School of Medicine, Suwon; d Department of Medicine/Cardiology, Cheil General Hospital, Kwandong University College of Medicine, Seoul, Korea.

**Keywords:** blood pressure, fimasartan, hypertension, hyperuricemia, metabolic syndrome

## Abstract

Previous epidemiological studies have suggested that uric acid is an independent risk factor for incident hypertension, whereas few studies have evaluated the effect of hyperuricemia on blood pressure control in hypertensive patients. We investigated whether hyperuricemia predicts uncontrolled hypertension through a large-scale prospective cohort study with hypertensive patients treated with fimasartan in the Republic of Korea (the Kanarb–Metabolic Syndrome study).

Of the 10,601 hypertensive patients who were recruited from 582 private clinics and 11 university hospitals at baseline, 7725 completed the follow-up after 3 months of fimasartan medication, and 6506 were included in the analysis after excluding those with missing values. We estimated the risk of uncontrolled hypertension after 3 months (≥130/80 mm Hg in those with diabetes or chronic renal failure and ≥140/90 mm Hg in the remaining patients) related with baseline hyperuricemia (serum uric acid ≥7 mg/dL in males ≥6 mg/dL in females) using multiple logistic regression models.

Hyperuricemia increased the risk of uncontrolled hypertension after 3 months of fimasartan medication (odds ratio, 1.247; 95% confidence interval, 1.063–1.462). Males in the highest quartile of uric acid level were at a 1.322 (95% confidence interval, 1.053–1.660) times higher risk of uncontrolled hypertension in reference to the lowest quartile; the same analyses in females were not significant. Patients without metabolic syndrome had significantly higher odds of uncontrolled hypertension with hyperuricemia (odds ratio, 1.328; 95% confidence interval, 1.007–1.751).

Hyperuricemia predicted uncontrolled hypertension even after 3 months of fimasartan treatment in hypertensive patients.

## Abstract 2

Supplemental Digital Content is available in the text

## 1 Introduction

Hypertension is one of the most challenging issues for public health. Its complications contribute to 9.4 million deaths among 17 million deaths from cardiovascular disease annually worldwide.^[1]^ In 2008, ∼40% of adults aged ≥25 years globally were diagnosed with hypertension,^[2]^ and in the Republic of Korea, the hypertension prevalences in males and females were 32.4% and 22.2%, respectively, in 2013.^[3]^

Although antihypertensive medications are considered effective for controlling blood pressure (BP), uncontrolled hypertension remains prevalent, with uncontrolled hypertension affecting an ∼1 billion people globally,^[4]^ 48.2% of hypertensive patients in the United States (2011–2012),^[5]^ and 57.5% of hypertensive patients in the Republic of Korea (2013).^[3]^ Uncontrolled hypertension refers to a lack of BP control due to poor compliance, insufficient drug regimen, or drug resistance.^[6]^ To prevent complications of hypertension such as coronary heart disease, stroke, and renal disease,^[7,8]^ risk factors for uncontrolled hypertension should be considered when establishing appropriate BP control strategies.

Despite long-standing controversy in the causal role of serum uric acid in hypertension and cardiovascular disease, there is a recently growing interest in serum uric acid as an independent risk factor for incident hypertension based on numerous prospective studies conducted in the United States, China, Italy, and Japan.^[9–15]^ A recent meta-analysis also reported a dose–response relationship between hyperuricemia and incident hypertension.^[16]^ This relation is supported by various animal experiments reporting that uric acid may inhibit endothelial function by suppressing endothelial nitric oxide synthase (NOS),^[17]^ directly influence proliferation and migration of vascular smooth muscle cells,^[18]^ activate the renin–angiotensin system (RAS), and reduce macula densa neuronal NOS in the kidney.^[19]^ According to one of these animal studies, decreasing uric acid levels reduced BP in rats with pre-existing hypertension.^[19]^ Moreover, in a clinical study, treatment of hyperuricemia was beneficial for lowering BP in adolescents with newly-diagnosed hypertension.^[20]^ Therefore, hyperuricemia might also induce high BP in hypertensive patients, ultimately increasing the risk of cardiovascular disease. In practice, high serum uric acid increased the risk of cardiovascular events in hypertensive patients despite treatment ;^[21,22]^ however, Alderman et al^[21]^ reported high-normal BP during the treatment (138.9/85.4 mm Hg), implying that many participants might still have had high BP, and Verdecchia et al^[22]^ did not report the BP during treatment. Thus, research on uncontrolled hypertension needs to be conducted to identify the relationship between hyperuricemia and cardiovascular events. To the best of our knowledge, studies of the effect of hyperuricemia on BP control in hypertensive patients have not been conducted.

In the present study, we investigated whether hyperuricemia increases the risk for uncontrolled hypertension using a large-scale prospective cohort with hypertension. Additionally, we examined whether persistent hyperuricemia increases the risk for uncontrolled hypertension.

## 2 Methods

### 2.1 Study participants

In the Kanarb–Metabolic Syndrome (K-MetS) study, which is described in detail elsewhere,^[23]^ the 10,601 hypertensive patients were administered fimasartan (Kanarb^®^, Boryung Pharmaceutical Company, Korea), which is an angiotensin receptor blocker. Briefly, the K-MetS study was a multicenter (582 private clinics and 11 university hospitals), prospective cohort study; all participants were ≥20 years old, had never used fimasartan medication at baseline, and started fimasartan use after the entry examination, which was performed between October 2011 and October 2012. Of the 10,601 participants, 7725 (72.9%) were followed up for 3 months after enrollment. After excluding participants with missing values, 6506 participants were included in the statistical analysis. The median hypertension duration was 1.35 years (25–75th percentile, 0.10–5.65), and 3911 (60.1%) patients used other antihypertensive drugs in conjunction with fimasartan, including other angiotensin receptor blockers (N = 1442, 22.2%) and calcium channel blockers (N = 1671, 25.7%). The distribution of hyperuricemia, age, sex, smoking, drinking, cardiovascular disease, dyslipidemia, impaired renal function, gamma-glutamyltranspeptidase (r-GTP), and medication compliance in the study subjects were not significantly different from those of the excluded participants (Table S1). The proportions of high waist circumference (54.6% vs 46.0%), diabetes (24.8% vs 19.4%), and obesity (50.5% vs 47.4%) were higher in the included participants than in the excluded participants, but the proportion of high BP at baseline (67.2% vs 74.5%) was lower.

This study was reviewed and approved by the Institutional Review Board Committee at the Cheil General Hospital, Kwandong University College of Medicine, on behalf of 582 primary clinics. The institutional review board committees of the remaining 10 hospitals approved this study, and informed consent was acquired from all participants.

### 2.2 Measurements

At the entry examination, we obtained information on smoking (current smoker, ex-smoker, or nonsmoker), alcohol consumption (drinker or nondrinker), and past medical history. At the 3-month follow-up, medication compliance was evaluated by counting pills taken by patients from the pills prescribed at baseline and was categorized as 100%, 90% to 99%, 80% to 89%, <80%, or 0%.

Systolic BP and diastolic BP measurements were performed with standardized methods, and BP at baseline was categorized into optimal (systolic BP <120 mm Hg and diastolic BP <80 mm Hg), normal (120–129 mm Hg and 80–84 mm Hg), high-normal (130–139 mm Hg and 85–89 mm Hg), or high (≥140 mm Hg or ≥90 mm Hg) BP according to the European Society of Hypertension guidelines.^[24]^ Uncontrolled hypertension was defined as systolic BP ≥130 or diastolic BP ≥80 in those with diabetes or chronic renal failure and systolic BP ≥140 or diastolic BP ≥90 in the rest of the participants.^[25]^ Body mass index (BMI) was classified as underweight (<18.5 kg/m^2^), normal (18.5–24.9 kg/m^2^), or obese (≥25.0 kg/m^2^).^[26]^ Waist circumference was measured at the midpoint between the ribs and iliac crest. A high waist circumference was defined as ≥90 cm in males or ≥80 cm in females.^[27]^

All blood samples were obtained after a ≥8-hour fast and were analyzed in a central laboratory (Green Cross Reference Lab, Republic of Korea). Blood tests included serum uric acid, r-GTP, fasting blood glucose, total cholesterol, triglycerides, high-density lipoprotein cholesterol (HDL-C), and low-density lipoprotein cholesterol (LDL-C). Hyperuricemia was defined as serum uric acid ≥7 mg/dL in males and ≥6 mg/dL in females,^[12,28]^ and we also categorized serum uric acid levels using quartiles, separately by sex. Dyslipidemia was defined as one of following: LDL-C ≥160 mg/dL, total cholesterol ≥240 mg/dL, HDL-C <40 mg/dL in male or <50 mg/dL in female or treated with oral medication. Underlying cardiovascular disease consisted of ischemic heart disease, atrial fibrillation, congestive heart failure, or stroke. Pre-existing diabetes was defined as diagnosis by a doctor or treated with medication. The glomerular filtration rate (GFR) was estimated using the Modification of Diet in Renal Disease (MDRD) formula, and the impaired renal function was identified as an estimated GFR < 60 mL/min/1.73 m^2^.

### 2.3 Statistical analysis

Baseline characteristics of those with uncontrolled hypertension were compared with those with controlled hypertension using *t* tests (age), Mann–Whitney tests (r-GTP), or chi-squared tests (categorical variables). The K-MetS study was a diseased cohort study with regular follow-up, and uncontrolled hypertension might have occurred before the follow-up examination; therefore, we used multiple logistic regression models for the left-censored data. We adjusted for age and sex in Model 1 and age, sex, smoking, alcohol consumption, high waist circumference, and BMI category in Model 2. Model 3 was adjusted for the Model 2 variables in addition cardiovascular disease, diabetes, dyslipidemia, log-transformed r-GTP, impaired renal function, systolic BP at baseline, diastolic BP at baseline, and medication compliance. In addition to using hyperuricemia at baseline or sex-separate quartiles of uric acid at baseline as an independent variable, we harnessed a 3-group variable related to persistent hyperuricemia, as follows: nonhyperuricemic at baseline, hyperuricemic at baseline but nonhyperuricemic at follow-up, and hyperuricemic at both baseline and follow-up. For posthoc subgroup analysis, we conducted stratified analyses according to the baseline prevalence of metabolic syndrome. Hypertensive patients with pre-existing metabolic syndrome might have a different baseline risk of uncontrolled hypertension because hyperuricemia might also cause metabolic syndrome^[29]^ and interact with metabolic syndrome components.^[30]^ The criteria for metabolic syndrome were the presence of ≥3 of the following 5 components: (1) BP ≥130/85 mm Hg or medicated; (2) fasting blood glucose ≥100 mg/mL or medicated; (3) waist circumference ≥90 cm for male or ≥80 cm for female, based on Asian-specific standards; (4) HDL-C <40 mg/dL for male or <50 mg/dL for female or medicated; and (5) triglycerides ≥150 mg/dL or medicated.^[27]^ We estimated the adjusted odds ratio (OR) and 95% confidence interval (95% CI). SAS version 9.3 (SAS Institute, Cary, NC) was used to conduct the statistical analyses.

## 3 Results

Of the 10,601 K-MetS study participants at baseline, 7725 completed the 3-month follow-up, and 6506 participants were analyzed after excluding those with missing values. Among the 6506 patients, 1005 (15.5%) had hyperuricemia (Table 1). The mean (standard deviation) age was 56.1 (10.5) years, and 51.9% were males. Cardiovascular disease, diabetes, and dyslipidemia were present in 352 (5.4%), 1094 (16.8%), and 3530 (54.3%) participants, respectively. After 3 months, 1925 (29.6%) participants had uncontrolled hypertension.

**Table 1:**
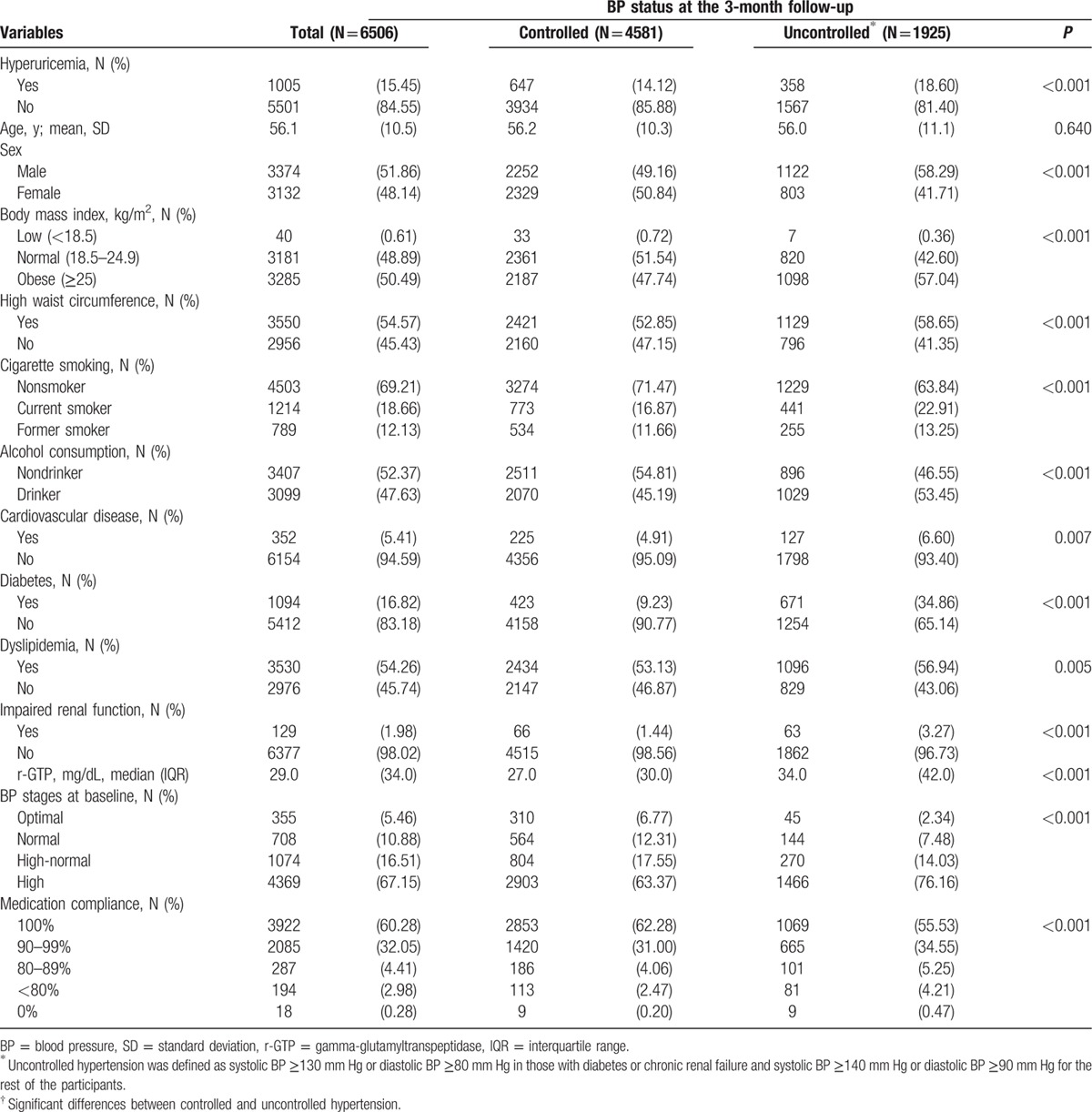
Baseline characteristics of study participants and blood pressure status 3 months after initiation of fimasartan medication.

In the patients with uncontrolled BP, 358 (18.6%) had hyperuricemia at baseline, and 647 (14.1%) of the patients with controlled BP had hyperuricemia (*P* < 0.001). The mean systolic and diastolic BPs at baseline were 143.89 (standard deviation, 17.04) mm Hg and 88.30 (11.22) mm Hg, respectively, and 67.2% of the participants had high BP at baseline. Males, obese participants, current smokers, drinkers, and those with cardiovascular disease, diabetes, impaired renal function, high BP at baseline, or lower compliance were more likely to have uncontrolled hypertension.

Hyperuricemia increased the risk for uncontrolled hypertension (OR, 1.247; 95% CI, 1.063–1.462) in Model 3 (Table 2). Based on the analysis using quartiles, the results in females were not significant, whereas males with the highest quartile of uric acid had a higher risk of uncontrolled hypertension in reference to the lowest quartile (OR, 1.322; 95% CI, 1.053–1.660), with borderline significance for the trend (*P* = 0.06).

**Table 2:**
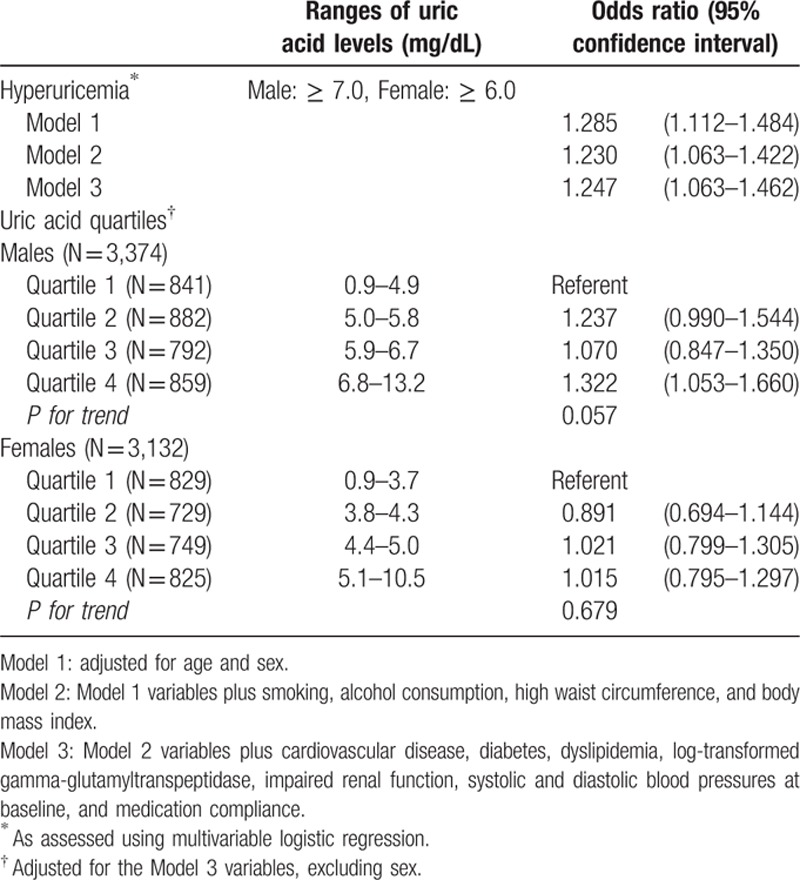
Serum uric acid levels and the risk of uncontrolled hypertension after 3 months of fimasartan medication.

Those who were hyperuricemic at both baseline and follow-up had an increased risk for uncontrolled hypertension at the 3-month follow-up compared with those who were nonhyperuricemic at baseline in Model 1 (OR, 1.267; 95% CI, 1.040–1.543; Table 3). However, the risks were not significant in Models 2 and 3.

**Table 3:**
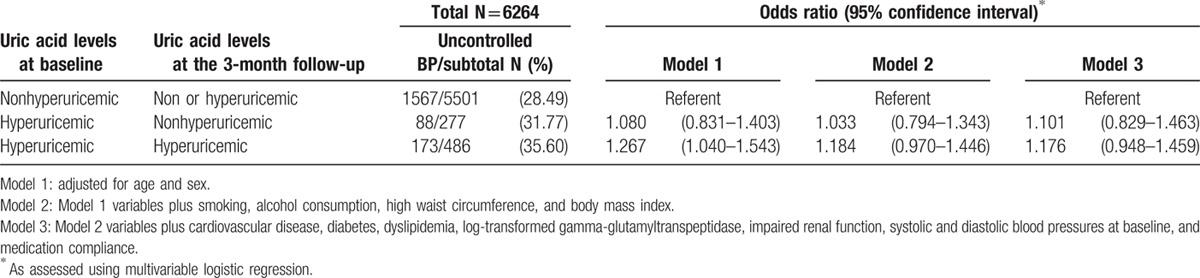
Persistent hyperuricemia and the risk of uncontrolled hypertension after 3 months of fimasartan medication.

Metabolic syndrome was present in 3670 participants, including 1259 patients with uncontrolled hypertension (34.3%; Table 4). Uncontrolled BP was present for 23.5% (n = 666/2836) of the participants without metabolic syndrome. In the multivariable analyses with Model 3, patients without metabolic syndrome had an increased risk of uncontrolled BP related with hyperuricemia (OR, 1.328; 95% CI, 1.007–1.751), whereas patients with metabolic syndrome did not (OR, 1.201; 95% CI, 0.988–1.461).

**Table 4:**
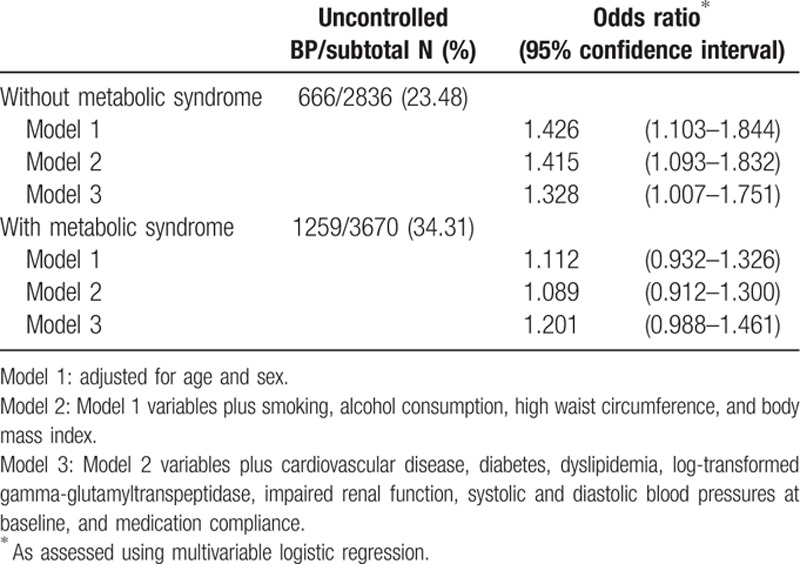
Hyperuricemia-induced risk of uncontrolled hypertension at the 3-month follow-up after stratification for metabolic syndrome.

## 4 Discussion

In this prospective cohort study, hyperuricemia increased the risk for uncontrolled hypertension after 3 months of medication with fimasartan in hypertensive patients. Although there are no studies linking hyperuricemia and uncontrolled hypertension in patients, many prospective studies have suggested the possible role of serum uric acid in the development of hypertension among normotensive individuals.^[9–14,16]^

Hyperuricemia suggests renal involvement including reduced nitric oxide synthesis, stimulation of the RAS, and microvascular and inflammatory changes,^[19,31,32]^ leading to the development of hypertension. In one of these studies, decreasing uric acid levels helped decrease BP in rats with pre-existing hypertension.^[19]^ Given the reported effectiveness of hyperuricemia treatment on BP control in hypertensive adolescents,^[20]^ it is plausible to suggest that hyperuricemia independently predicts uncontrolled hypertension in hypertensive patients. The findings of the present study indicate that hyperuricemic patients with hypertension might have uncontrolled BP despite successful antihypertensive medication, which is clinically relevant. Moreover, persistent hyperuricemia significantly increased the risk of uncontrolled hypertension in an age- and sex-adjusted statistical model; however, this relationship was no longer significant after further adjustment. Because of the observational nature of this study, we cannot conclude that correcting hyperuricemia is beneficial for BP control in hypertensive patients; however, uncorrected hyperuricemia might negatively influence BP status among treated hypertensive patients in a real-world setting. Although metabolic syndrome itself can lead to uncontrolled hypertension,^[33,34]^ hyperuricemia can also increase the risk of uncontrolled BP in hypertensive patients without metabolic syndrome, as evidenced by the present findings.

Because a universal cut-off for hyperuricemia has not been established, previous studies of the association between uric acid and incident hypertension used various definitions of hyperuricemia. To prevent possible misclassification and identify a dose–response relationship, we also conducted analyses using quartiles of uric acid levels. As a result, we did not observe a dose–response relationship, although one was suggested in some prospective studies^[9,13]^ and a meta-analysis^[16]^ of uric acid and new-onset hypertension. One possible explanation is the difference in study participants; previous studies included normotensive participants, whereas we included hypertensive participants. Compared with normotensive individuals, hypertensive patients are more likely to have underlying renal microvascular injury. After renal injury, increases in BP may no longer rely on uric acid levels.^[35]^ Thus, a dose–response relationship between uric acid and high BP might be difficult to observe in hypertensive patients. Similarly, Feig and Johnson^[36]^ suggested that early hypertension is associated with high serum uric acid and high serum renin levels, leading to higher responsiveness to RAS blockers. In our further stratified analyses, ≥40 year-old patients were at a significantly higher risk of uncontrolled hypertension based on hyperuricemia (N = 6,150; OR, 1.215; 95% CI, 1.029–1.436). In the younger subjects, the risk was higher but insignificant (N = 356; OR, 1.659; 95% CI, 0.911–3.020), which might be due to a greater efficacy of fimasartan or a small sample size.

The K-MetS study population consisted of hypertensive patients with a higher proportion of metabolic syndrome than the general population.^[23]^ As expected, the proportion of uncontrolled hypertension was higher in those with metabolic syndrome. However, there was a significant risk of uncontrolled BP due to hyperuricemia in those without metabolic syndrome, but not in those with metabolic syndrome. It is difficult to explain this with existing mechanisms, but there are several possible explanations. The causal role of uric acid in high BP may involve several pathways: NOS-related endothelial dysfunction, RAS activation, and stimulated proliferation of vascular smooth muscle cells.^[17–19]^ The NOS- and RAS-related mechanisms seem to overlap with those of metabolic syndrome leading to high BP. Metabolic syndrome may be related to the development of hypertension via complex pathways including an increase in angiotensin II via adiposity-induced inflammation and a decrease in NOS activation via oxidative stress and insulin resistance.^[37]^ The two mechanisms are probably intertwined in coexisting hyperuricemia and metabolic syndrome, in which the effect of hyperuricemia on uncontrolled BP might be undetectable, as shown in our study. Moreover, angiotensin receptor blockers have several effects beyond BP reduction. The possible effect of lowering uric acid levels^[38]^ might attenuate the risk of uncontrolled hypertension related with hyperuricemia. Fimasartan reduces RAS activation, oxidative stress, and renal inflammation,^[39]^ which might intervene in the pathways from hyperuricemia or metabolic syndrome to uncontrolled BP. Regarding the other mechanisms related with stimulated proliferation of vascular smooth muscle cells, animal experiments suggest that fimasartan might inhibit vascular smooth muscle cell proliferation after high-glucose administration^[40]^ and a higher proportion of the cells in atherosclerotic plaque after injury.^[41]^ Nonetheless, there is no evidence to date to support that fimasartan administration depresses the vascular smooth muscle cell proliferation induced by hyperuricemia. Additional biological evidence is required to understand the shared pathophysiology of hyperuricemia, metabolic syndrome, and hypertension.

Despite the many prospective studies relating serum uric acid with new-onset hypertension, the present study is the first, to our knowledge, to suggest that hyperuricemia predicts uncontrolled hypertension after only 3 months of treatment. However, the present study has several limitations that should be considered. First, our results might not generalize to all hypertensive patients. Because the K-MetS study recruited hypertensive patients who were willing to start a new agent, it is possible that some patients already had predisposing factors for uncontrolled hypertension, potentially supported by the 67.2% of patients with high BP at baseline. However, after 3 months of fimasartan administration, the proportion of uncontrolled hypertension was 29.6%, much lower than the reported prevalence (57.5%) in the Republic of Korea.^[3]^ This indicates that adherence to medication has a strong effect on BP control. When we restricted the analysis to the subjects with good adherence (≥80%),^[42]^ the association between hyperuricemia and uncontrolled hypertension remained significant (OR, 1.237; 95% CI, 1.051–1.455) in Model 3. Therefore, the present study may be the most available setting to suggest that hyperuricemia in hypertensive patients predicts uncontrolled hypertension despite good adherence to antihypertensive medication. Second, we could not obtain information on underlying gout and related medications. Some patients with hyperuricemia have a history of gout, with or without treatment. Even among patients with normal uric acid levels, some patients might be using medications that decrease serum uric acid levels. To estimate a more valid risk for uncontrolled hypertension with hyperuricemia, further investigations that consider the history of gout and related medications are necessary. Finally, the proportions of included and excluded participants with a high waist circumference, diabetes, or obesity were different. The included participants had higher proportions of the cardiovascular risk factors, reflected in the higher number of patients with metabolic syndrome (56.4% vs 33.5%). Regarding the weak and insignificant association between hyperuricemia and uncontrolled hypertension in those with metabolic syndrome, the inclusion of more patients with metabolic syndrome is more likely to underestimate the true risk. Similarly, fewer participants lost to follow-up had a high waist circumference (47.7%), obesity (46.5%), or diabetes (12.9%) than the participants who completed the follow, which might tend toward null findings.

The findings of the present study suggest that hyperuricemia significantly predicts uncontrolled hypertension in hypertensive patients after 3 months of fimasartan treatment. Hyperuricemic patients with hypertension are more likely to have uncontrolled BP despite successful treatment with antihypertensive agents. In particular, the risk of uncontrolled BP related with hyperuricemia was prominent in hypertensive patients without metabolic syndrome. Uric acid levels need to be considered in strategies for BP control in hypertensive patients, even with good adherence to antihypertensive medications.

## Supplementary Material

Supplemental Digital Content
