# Hyperuricemia and uncontrolled hypertension in treated hypertensive patients K-MetS Study: Erratum

**DOI:** 10.1097/01.md.0000503535.96686.ae

**Published:** 2016-10-07

In the article “Hyperuricemia and uncontrolled hypertension in treated hypertensive patients K-MetS Study”,^[1]^ which appeared in Volume 95, Issue 28 of *Medicine*, Dr. Park's affiliation was originally given incorrectly. The article has since been corrected online.
