# Supplementary material for: Hyperuricemia and uncontrolled hypertension in treated hypertensive patients: K-MetS Study
Source: Medicine (Baltimore). 2016 Jul 18;95(28):e4177. doi: 10.1097/MD.0000000000004177 (PMC4956806; doi:10.1097/MD.0000000000004177)
Supplement: Supplemental Digital Content [file medi-95-e4177-s001.docx]

**ONLINE SUPPLEMENT**

**Title**

Hyperuricemia and Uncontrolled Hypertension in Treated Hypertensive Patients: Kanarb-Metabolic Syndrome (K-MetS) Study

**Authors**

Jaelim Cho, Changsoo Kim, Dae Ryong Kang, Jeong Bae Park

**Contents**

Supplementary Table 1. Comparisons of baseline characteristics between included and excluded participants

| **Table S1. Comparisons of baseline characteristics between included and excluded participants** | | | | | | |
| --- | --- | --- | --- | --- | --- | --- |
|  |  | **Study participants completing three months follow-up (N = 7,725)** | | | | |
| **Variables** |  | **Included**  **(N = 6,506)** | | **Excluded**  **(N = 1,219)** | | **p^*^** |
| Hyperuricemia, N (%) |  | 1,005 | (15.45) | 109 | (16.05) | 0.719 |
|  | Missing |  |  | 540 |  |  |
| Age (years), mean (SD) |  | 56.1 | (10.5) | 56.6 | (11.2) | 0.169 |
| Sex, N (%) | Male | 3,374 | (51.86) | 629 | (51.60) | 0.892 |
|  | Female | 3,132 | (48.14) | 590 | (48.40) |  |
| Body mass index (kg/m^2^), N (%) | Low (<18.5) | 40 | (0.61) | 14 | (1.15) | 0.024 |
|  | Normal (18.5−24.9) | 3,181 | (48.89) | 626 | (51.44) |  |
|  | Obese (≥25) | 3,285 | (50.49) | 577 | (47.41) |  |
|  | Missing |  |  | 2 |  |  |
| High waist circumference, N (%) |  | 3,550 | (54.57) | 543 | (45.98) | <0.001 |
|  | Missing |  |  | 38 |  |  |
| Cigarette smoking, N (%) | Non-smoker | 4,503 | (69.21) | 547 | (67.78) | 0.311 |
|  | Current smoker | 1,214 | (18.66) | 147 | (18.22) |  |
|  | Former smoker | 789 | (12.13) | 113 | (14.00) |  |
|  | Missing |  |  | 412 |  |  |
| Alcohol consumption, N (%) | Non-drinker | 3,407 | (52.37) | 412 | (52.96) | 0.785 |
|  | Drinker | 3,099 | (47.63) | 366 | (47.04) |  |
|  | Missing |  |  | 441 |  |  |
| Cardiovascular disease, N (%) |  | 352 | (5.41) | 69 | (5.66) | 0.776 |
| Diabetes, N (%) |  | 1,094 | (16.82) | 155 | (12.72) | <0.001 |
| Dyslipidemia, N (%) |  | 3,530 | (54.26) | 400 | (55.48) | 0.559 |
|  | Missing |  |  | 498 |  |  |
| Impaired renal function, N (%) |  | 129 | (1.98) | 6 | (0.88) | 0.063 |
|  | Missing |  |  | 540 |  |  |
| r-GTP (mg/dL), median (IQR) |  | 29.0 | (34.0) | 28.0 | (36.0) | 0.843 |
|  | Missing, N (%) |  |  | 540 |  |  |
| BP stages at baseline, N (%) | Optimal | 355 | (5.46) | 48 | (3.94) | <0.001 |
|  | Normal | 708 | (10.88) | 91 | (7.47) |  |
|  | High-normal | 1,074 | (16.51) | 172 | (14.11) |  |
|  | High | 4,369 | (67.15) | 908 | (74.49) |  |
| Medication compliance, N (%) | 100% | 3,922 | (60.28) | 642 | (59.17) | 0.116 |
|  | 90-99% | 2,085 | (32.05) | 368 | (33.92) |  |
|  | 80-89% | 287 | (4.41) | 54 | (4.98) |  |
|  | <80% | 194 | (2.98) | 20 | (1.84) |  |
|  | 0% | 18 | (0.28) | 1 | (0.09) |  |
|  | Missing |  |  | 134 |  |  |
| BP: blood pressure, SD: standard deviation, r-GTP: gamma-glutamyltranspeptidase, IQR: interquartile range  ^*^ Significant differences between included and excluded participants | | | | | | |
